# Optimal PEEP with lowest (least injurious) transpulmonary driving pressure can be determined by a rapid two-PEEP-step procedure without esophageal pressure

**DOI:** 10.1186/s13054-022-04240-5

**Published:** 2022-11-29

**Authors:** O. Stenqvist

**Affiliations:** Sahlgrenska Academy, Gothenburg University, Gothenburg, Sweden

To the Editor,

In the recent study on personalized optimal PEEP in hypoxemic patients during pressure support ventilation (PSV), Slobod and coworkers assessed collapse and overdistension by electric impedance tomography (EIT) during a PEEP trial [1]. Normally, this assessment is based on airway driving pressure (ΔPAW) and respiratory system compliance, but in this study, lung compliance (CL) and transpulmonary pressure by esophageal pressure were the basis of the analysis. They showed that the PEEP level with a balance between collapse and overdistension tended to coincide with the level where *lung* compliance is highest. Consequently, transpulmonary driving pressure (ΔPL) was lowest at this PEEP level, requiring the lowest inspiratory effort during PSV.

The authors should be commended for the important step to exchange respiratory system compliance and airway driving pressure for *lung* compliance and *transpulmonary* driving pressure because transpulmonary pressure is the pressure that directly affects lung tissue. In addition, it has been shown that the PEEP level with lowest airway driving pressure not necessarily coincides with the PEEP level with lowest transpulmonary driving pressure [2, 3].

Protective ventilation requires assessment of lung mechanics and optimal PEEP as early as possible after start of mechanical ventilation. It is unlikely that a method encompassing a time-consuming multi-PEEP step trial, EIT and esophageal pressure measurements will gain wide clinical acceptance, especially not very early in the course of ventilator treatment or in the operating theater.

However, the PEEP level where transpulmonary driving pressure is lowest can be determined by a rapid two-PEEP-step procedure without both EIT and esophageal pressure measurements [2, 3]. This method, the PEEP step method, is based on the fact that the change in end-expiratory lung volume (ΔEELV) following a PEEP change is determined by the size of the PEEP step (ΔPEEP) and the elastic properties of the lung only, ΔEELV = ΔPEEP × CL, i.e., the chest wall does not impede PEEP inflation. *(Details of the determinants of *Δ*EELV in e-supplement).* This is an effect of the chest wall striving outwards to a higher volume, 70–80% of total lung capacity during expirations, not only at functional residual capacity, but also at increased EELV and PEEP. If ΔEELV is measured as the cumulative difference in expiratory tidal volume (VT) between PEEP levels [4], lung compliance can be calculated, CL_PSM_ = ΔEELV/ΔPEEP and transpulmonary driving pressure, ΔPL = VT/CL_PSM_. Also, during PEEP inflation:

- Transpulmonary pressure increases as much as PEEP is increased.
- Transpulmonary driving pressure of a tidal volume equal to ΔEELV is equal to ΔPEEP.
- Transpulmonary pressure at a certain lung volume is the same irrespective of whether this volume has been reached by tidal inflation or PEEP inflation, i.e., end-inspiratory transpulmonary plateau pressure from low PEEP level is equal to end-expiratory transpulmonary pressure of the high PEEP level.

Because of these features of PEEP inflation, a lung P/V curve can be calculated by a two-PEEP-step procedure from baseline PEEP to end-inspiration at the highest PEEP level. The PEEP level where transpulmonary driving pressure is lowest (least injurious) can then be calculated as the PEEP level half a tidal volume below the steepest point of the lung P/V curve.

Slobod and coworkers did not determine ΔEELV in their study, but it is possible to illustrate the performance of the PEEP step method by calculating ΔEELV as ΔPEEP x CL between PEEP levels. This enabled the calculation of the complete lung P/V curve and optimal PEEP in the two extreme patients, patients 1 and 3 (Fig. 1). (For details, See Additional file 1: e-supplement.)

**Fig. 1:**
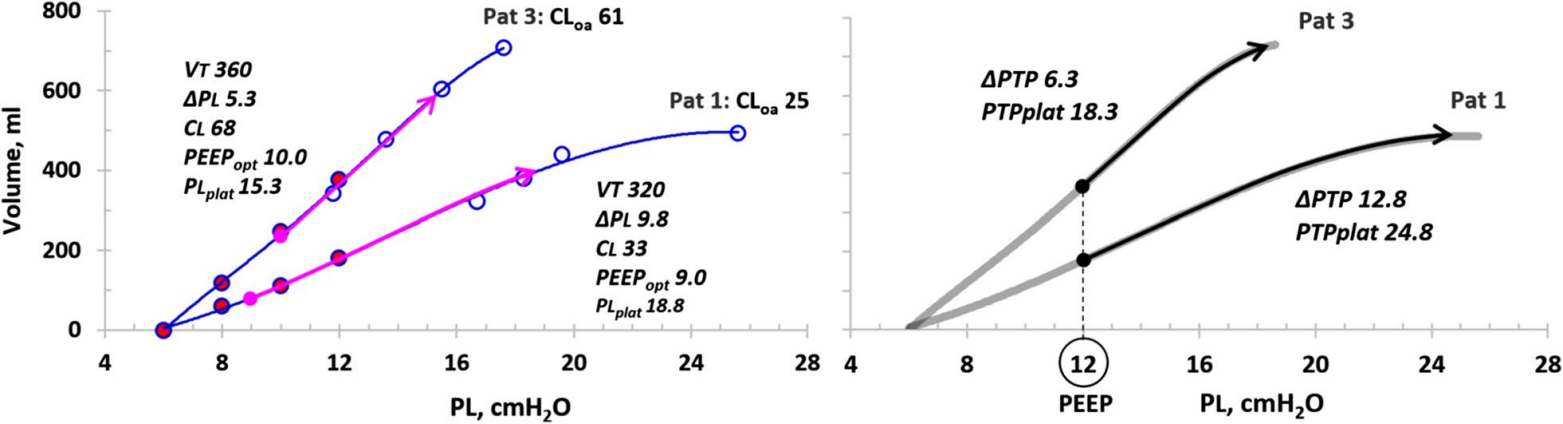
*Left panel*: Lung P/V curves of patients 1 and 3 with overall lung compliance of 25 and 61 ml/cmH_2_O respectively. Overall lung compliance was calculated as end-inspiratory lung volume at the highest PEEP level (12 cmH_2_O) divided by end-inspiratory transpulmonary pressure at the highest PEEP level minus 6 cmH_2_O (lowest PEEP level). Circles with red filling: end-expiratory transpulmonary pressure (= PEEP). Circles without filling: end-inspiratory transpulmonary pressure. Magenta arrows: tidal lung P/V curves at optimal PEEP obtained by graphical plotting from the lung P/V curve. Data of optimal PEEP tidal volume in italics: VT = tidal volume, ΔPL = transpulmonary driving pressure, CL = lung compliance, PEEP_opt_ = optimal PEEP level, PL_plat_ = end-inspiratory transpulmonary pressure. *Right panel*: Light gray lung P/V curves of patients 1 and 3 can be used as clinical decision support as a tidal lung P/V curve with any combination of PEEP and VT will be positioned on the complete lung P/V curve. This makes it possible to estimate the effect of changes in PEEP and tidal volume. In this case, the tidal lung P/V curves (black arrows) are depicted starting from PEEP 12 cmH_*2*_O. In patient 3 with a moderately decreased overall lung compliance to 61 ml/cmH_*2*_O (“normal” lung compliance 90–110 ml/cmH_*2*_*O *[3]), transpulmonary driving pressure increases marginally and remains within safe limit, i.e., the patient responds favorably to PEEP. In patient 1, an increase in PEEP to 12 cmH_*2*_O results in a ΔPL 40% higher almost 13 cmH_*2*_O than at PEEP 9 cmH_*2*_O with significant risk of ventilation induced lung injury

There is no safety limit determined for ΔPL, but it can be deduced from the fact that the upper safety limit for ΔPAW is 15 cmH_2_O and average ratio of ΔPL/ΔPAW is 0.70, which results in a safety limit for ΔPL of 0.7 × 15 ≈ 10 cmH_2_O [2, 5]. Patient 1 has an overall lung compliance of 25 ml/cmH_2_O, which is even lower than reported in severe ARDS [5]. Transpulmonary driving pressure at optimal PEEP is almost 10 cmH_2_O in spite of a low tidal volume (320 ml). A PEEP increase to 12 cmH_2_O would result in a ΔPL of 12.8 cmH_2_O and an end-inspiratory transpulmonary plateau pressure of 25 cmH_2_O, both dangerously high. In patient 3, with a moderately lowered overall lung compliance, a PEEP increase to 12 cmH_2_O only causes a small increase in ΔPL to 6.3 cmH_2_O and a transpulmonary plateau pressure of 18 cmH_2_O, both well within the safety limits (Fig. 1).

In summary, a complete lung P/V curve and optimal PEEP with lowest transpulmonary driving pressure can be determined by a rapid two-PEEP-step procedure, where ΔEELV is determined by the ventilator pneumotachograph. Neither esophageal pressure nor EIT is required. The lung P/V curve can be used as clinical decision support to estimate the effect of changes in PEEP and tidal volume on transpulmonary driving and plateau pressure.

## Supplementary Information

**Additional file 1.** E-supplement.

## Data Availability

Not applicable.

## References

[1] Slobod D, Leali M, Spinelli E, Grieco DL, Spadaro S, Mauri T (2022). Integrating electrical impedance tomography and transpulmonary pressure monitoring to personalize PEEP in hypoxemic patients undergoing pressure support ventilation. Crit Care.

[2] Grivans C, Stenqvist O. Gas distribution by EIT during PEEP inflation: PEEP response and optimal PEEP with lowest trans-pulmonary driving pressure can be determined without esophageal pressure during a rapid PEEP trial in patients with acute respiratory failure. Physiol Meas; 2022. 10.1088/1361-6579/ac8ccc 36007512

[3] Persson P, Stenqvist O (2022). Protective positive end-expiratory pressure and tidal volume adapted to lung compliance determined by a rapid positive end-expiratory pressure-step procedure in the operating theatre: a post hoc analysis. Br J Anaesth.

[4] Grivans C, Lundin S, Stenqvist O, Lindgren S (2011). Positive end-expiratory pressure-induced changes in end-expiratory lung volume measured by spirometry and electric impedance tomography. Acta Anaesthesiol Scand.

[5] Gattinoni L, Pelosi P, Suter PM, Pedoto A, Vercesi P, Lissoni A (1998). Acute respiratory distress syndrome caused by pulmonary and extrapulmonary disease: different syndromes?. Am J Respir Crit Care Med.

