# Supplementary material for: Optimal PEEP with lowest (least injurious) transpulmonary driving pressure can be determined by a rapid two-PEEP-step procedure without esophageal pressure
Source: Crit Care. 2022 Nov 29;26:370. doi: 10.1186/s13054-022-04240-5 (PMC9710035; doi:10.1186/s13054-022-04240-5)
Supplement: Supplementary file 1 — Additional file 1. E-supplement. [file 13054_2022_4240_MOESM1_ESM.docx]

E-supplement of

**Optimal PEEP with lowest (least injurious) transpulmonary pressure can be determined by a rapid two-PEEP-step procedure without esophageal pressure**

O. Stenqvist

**The determinants of ΔEELV**

The change in end-expiratory lung volume (ΔEELV) following a PEEP change (ΔPEEP) is determined by the size of the PEEP step and the elastic properties of the lung only. ΔEELV = CL x ΔPEEP. This is verified by a comparison of ΔEELV measured by the ventilator pneumotachograph and ΔEELV calculated as CL x ΔPEEP. In studies by Gattinoni, Pelosi, Garnero, Lundin and Persson data of measured ΔEELV and CL determined by esophageal pressure were found, which could be used for the correlation calculations [1-5] .

*Fig. 1S. Cumulative ΔEELV was calculated as the change in PEEP divided by the lung elastance, ΔPEEP/ ΔEELV, where lung elastance was determined as the transpulmonary driving pressure divided by the tidal volume. Transpulmonary driving pressure was obtained using esophageal pressure measurements.(ΔPAW – ΔPES)/ VT. In the studies by Gattinoni et al [2] and Pelosi et al [4] PEEP steps were performed from ZEEP to respective PEEP level and back, 0-5-0, 0-10-0, 0-15-0 cmH_2_O in random order. The increase in end-expiratory lung volume (ΔEELV) between two PEEP levels was measured as the difference in volume exhaled from end-inspiration at PEEP to ZEEP during a prolonged expiration. In the Garnero et al study [1] PEEP steps was performed consecutively, starting at a PEEP of 5 cmH_2_O, 5-10-15-20-25-30-35-40 cmH_2_O. In the Lundin, Garnero and Persson studies, ΔEELV was measured as the cumulative difference in inspiratory and expiratory tidal volume between pressure/volume (P/V) equilibrium of two PEEP levels [6] (figure and figure text from [7] with permission from Intens Care Med).*

Calculation of the lung P/V curve

As the study by Slobod et al did not encompass direct information on tidal volumes or changes in end-expiratory lung volume, these were calculated from data in the study of lung compliance and transpulmonary driving pressure in table 1. Thus, tidal volume was calculated as lung compliance times transpulmonary driving pressure at each PEEP level:

VT = CL x ΔPL

The change in end-expiratory lung volume between PEEP levels were calculated as the change in PEEP times lung compliance:

**ΔEELV = ΔPEEP x CL**

Consequently

CL = ΔEELV/ΔPEEP

As the transpulmonary pressure increases in relation to the inflated volume and the elastic properties of and the lung, the end-expiratory transpulmonary pressure (PLEE) after a PEEP increase. increases with

ΔPLEE = ΔEELV/CL

and as

ΔPEEP = ΔEELV/CL.

the end-expiratory transpulmonary pressure increases as much as PAWEE is increased:

**ΔPLEE = ΔPEEP**

Based on this mathematical derivation of PEEP inflation, a table of airway, transpulmonary and esophageal pressure and corresponding lung volume was calculated for patient 3 of the Slobod study:

|  | **PAW** | **PL** | **PES** | **VOL** |
| --- | --- | --- | --- | --- |
| ***EE 6*** | 6.0 | 6.0 | 0.0 | 0 |
| ***EI 6*** | 15.0 | 11.8 | 3.2 | 342 |
| ***EE 8*** | 8.0 | 8.0 | 0.0 | 118 |
| ***EI 8*** | 16.8 | 13.6 | 3.2 | 476 |
| ***EE 10*** | 10.0 | 10.0 | 0.0 | 246 |
| ***EI 10*** | 18.7 | 15.5 | 3.2 | 604 |
| ***EE 12*** | 12.0 | 12.0 | 0.0 | 376 |
| ***EI 12*** | 20.7 | 17.6 | 3.1 | 706 |

*Table S1. Airway (PAW), transpulmonary (PL), and esophageal pressure (PES) at lung volumes from PEEP 6 cmH_2_O. EE = end-expiration, EI = end-inspiration. Note that end-expiratory transpulmonary pressure is equal to end-expiratory airway pressure (PEEP).*

*(for details of mathematical derivation and proof of concept of PEEP step method see Persson & Stenqvist in Br J Anaesth 2022 and Grivans & Stenqvist in Physiol Meas 2022 [8, 9])*

*
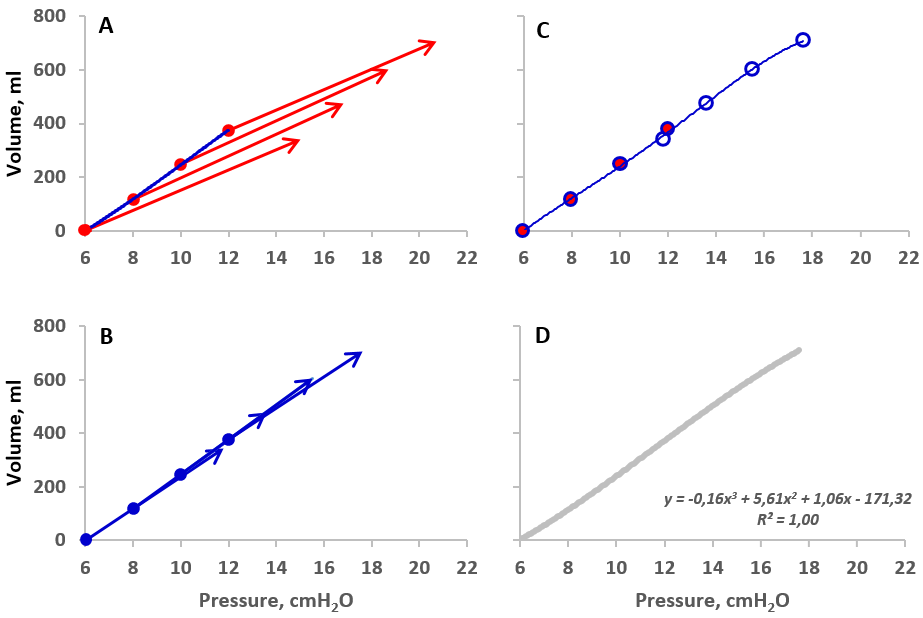
*

*Fig. 2S.* ***Panel A:*** *tidal airway P/V curves depicted starting from respective EELV level. Blue line is the end-expiratory airway pressure/end-expiratory lung volume P/V curve, which is equal to the lung P/V curve.* ***Panel B:*** *Tidal transpulmonary P/V curves depicted starting from respective end-expiratory lung volume level.* ***Panel C:*** *A best fit lung P/V curve is determined from end-expiration at baseline PEEP of 6 cmH_2_O to end-inspiration at the highest PEEP level, 12 cmH_2_O. Circles with red filling = end-expiratory P/V points. Open circles = end-inspiratory P/V points. Note that end-expiratory and end-inspiratory transpulmonary (lung) P/V points are aligned on a common P/V curve.* ***Panel D:*** *The complete lung P/V curve on which a tidal lung P/V curve with any combination of PEEP and tidal volume is postioned. This makes it possible to estimate the effect on transpulmonary driving pressure of changes in PEEP and tidal volume, i.e. it can be used for clinical decision support.*

The equation for the P/V curve (panel D of figure 2) show that the end-expiratory lung volume between ZEEP and PEEP 6 cmH_2_O is 171 ml. If this volume is added to table 1, an extrapolated table is obtained (Table S2):

|  | **PAW** | **PL** | **PES** | **VOL** | **VOL +** |
| --- | --- | --- | --- | --- | --- |
| ***EE 0*** | 0 | 0 | 0 |  | 0 |
| ***EE 6*** | 6,0 | 6,0 | 0,0 | 0 | 171 |
| ***EI 6*** | 15,0 | 11,8 | 3,2 | 342 | 513 |
| ***EE 8*** | 8,0 | 8,0 | 0,0 | 118 | 289 |
| ***EI 8*** | 16,8 | 13,6 | 3,2 | 476 | 647 |
| ***EE 10*** | 10,0 | 10,0 | 0,0 | 246 | 417 |
| ***EI 10*** | 18,7 | 15,5 | 3,2 | 604 | 775 |
| ***EE 12*** | 12,0 | 12,0 | 0,0 | 376 | 547 |
| ***EI 12*** | 20,7 | 17,6 | 3,1 | 706 | 877 |

*Table S2. Data extrapolated to ZEEP/FRC by adding the volume between PEEP 6 cmH2O and ZEEP, 171 ml in volume column VOL +.*

The extrapolated lung P/V curve is plotted in figure 3.

*
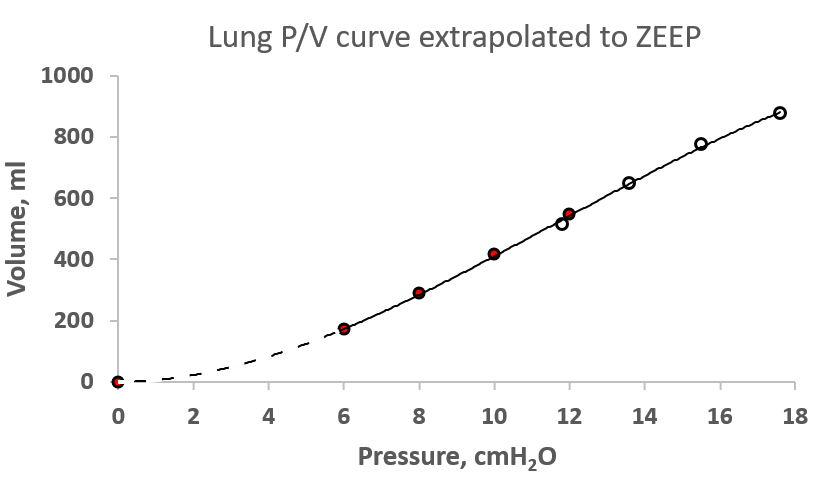
*

*Fig. 3S. Extrapolated lung P/V curve with prominent lower inflection zone.*

**The PEEP step method** is based on measurement of ΔEELV by the pneumotachograph of the ventilator by the cumulative expiratory tidal volume difference between PEEP levels [6]. This makes it possible to determine lung compliance:

CL_PSM_ = ΔEELV/ΔPEEP

and transpulmonary driving pressure:

ΔPL = VT/CL_PSM_

Calculation of optimal PEEP level is described in detail in the study by Grivans and Stenqvist, 2022 [8].

The method is validated, mathematically derived and proved and described in detail in the following articles: [3, 5, 6, 9-19]

**References**

1. Garnero A, Tuxen D, Ducros L, Demory D, Donati SY, Durand-Gasselin J, Cooper J, Hodgson C, Arnal JM: **Non-invasive assessment of lung elastance in patients with acute respiratory distress syndrome**. *Minerva anestesiologica* 2015, **81**(10):1096-1104.

2. Gattinoni L, Pelosi P, Suter PM, Pedoto A, Vercesi P, Lissoni A: **Acute respiratory distress syndrome caused by pulmonary and extrapulmonary disease. Different syndromes?** *American journal of respiratory and critical care medicine* 1998, **158**(1):3-11.

3. Lundin S, Grivans C, Stenqvist O: **Transpulmonary pressure and lung elastance can be estimated by a PEEP-step manoeuvre**. *Acta anaesthesiologica Scandinavica* 2015, **59**(2):185-196.

4. Pelosi P, Cereda M, Foti G, Giacomini M, Pesenti A: **Alterations of lung and chest wall mechanics in patients with acute lung injury: effects of positive end-expiratory pressure**. *American journal of respiratory and critical care medicine* 1995, **152**(2):531-537.

5. Persson P, Stenqvist O, Lundin S: **Evaluation of lung and chest wall mechanics during anaesthesia using the PEEP-step method**. *British journal of anaesthesia* 2018, **120**(4):860-867.

6. Grivans C, Lundin S, Stenqvist O, Lindgren S: **Positive end-expiratory pressure-induced changes in end-expiratory lung volume measured by spirometry and electric impedance tomography**. *Acta anaesthesiologica Scandinavica* 2011, **55**(9):1068-1077.

7. Stenqvist O: **Transpulmonary driving pressure, without esophageal pressure measurements, instead of airway driving pressure**. *Intensive care medicine* 2020, **46**(11):2113-2114.

8. Grivans C, Stenqvist, O.: **Gas distribution by EIT during PEEP inflation: PEEP response and optimal PEEP with lowest trans-pulmonary driving pressure can be determined without esophageal pressure during a rapid PEEP trial in patients with acute respiratory failure**. *Physiological measurement* 2022.

9. Persson P, Stenqvist O: **Protective positive end-expiratory pressure and tidal volume adapted to lung compliance determined by a rapid positive end-expiratory pressure-step procedure in the operating theatre: a post hoc analysis**. *British journal of anaesthesia* 2022, **128**(4):e284-e286.

10. Grivans C, Stenqvist O: **Gas distribution by EIT during PEEP inflation: PEEP response and optimal PEEP with lowest trans-pulmonary driving pressure can be determined without esophageal pressure during a rapid PEEP trial in patients with acute respiratory failure**. *Physiological measurement* 2022.

11. Grivans C LS, Stenqvist O: **Lung elastance can be determined without oesophageal pressure measurements**. *Critical care* 2013, **17**(Suppl 2):P130.

12. Lundin S, Stenqvist O: **Transpulmonary pressure can be determined without esophageal pressure measurements**. *Minerva anestesiologica* 2016, **82**(1):119-120.

13. Persson P, Lundin S, Stenqvist O: **Transpulmonary and pleural pressure in a respiratory system model with an elastic recoiling lung and an expanding chest wall**. *Intensive care medicine experimental* 2016, **4**(1):26.

14. Stenqvist O, Grivans C, Andersson B, Lundin S: **Lung elastance and transpulmonary pressure can be determined without using oesophageal pressure measurements**. *Acta anaesthesiologica Scandinavica* 2012, **56**(6):738-747.

15. Stenqvist O, Lundin S: **Lung elastance and transpulmonary pressure may be determined without using esophageal pressure measurements**. *American journal of respiratory and critical care medicine* 2014, **190**(1):120.

16. Stenqvist O, Persson P, Lundin S: **Can we estimate transpulmonary pressure without an esophageal balloon?-yes**. *Ann Transl Med* 2018, **6**(19):392.

17. Stenqvist O, Persson P, Stahl CA, Lundin S: **Monitoring transpulmonary pressure during anaesthesia using the PEEP-step method**. *British journal of anaesthesia* 2018, **121**(6):1373-1375.

18. Persson P, Ahlstrand R, Gudmundsson M, de Leon A, Lundin S: **Detailed measurements of oesophageal pressure during mechanical ventilation with an advanced high-resolution manometry catheter**. *Critical care* 2019, **23**(1):217.

19. Persson P, Stenqvist O, Lundin S: **Intraoperative measurement of transpulmonary pressure without an esophageal balloon catheter**. *American journal of respiratory and critical care medicine* 2017, **195:A3020**.
